# Bilateral Nerve Alterations in a Unilateral Experimental Neurotrophic Keratopathy Model: A Lateral Conjunctival Approach for Trigeminal Axotomy

**DOI:** 10.1371/journal.pone.0070908

**Published:** 2013-08-14

**Authors:** Takefumi Yamaguchi, Aslihan Turhan, Deshea L. Harris, Kai Hu, Harald Prüss, Ulrich von Andrian, Pedram Hamrah

**Affiliations:** 1 Schepens Eye Research Institute, Department of Ophthalmology, Harvard Medical School, Boston, Massachusetts, United States of America; 2 Cornea Service, Massachusetts Eye and Ear Infirmary, Department of Ophthalmology, Harvard Medical School, Boston, Massachusetts, United States of America; 3 Department of Neurology, Charité University Medicine, Berlin, Germany; 4 Immune Disease Institute, Program in Cellular and Molecular Medicine at Children's Hospital Boston, Harvard Medical School, Boston, Massachusetts, United States of America; University of Florida, United States of America

## Abstract

To study bilateral nerve changes in a newly developed novel mouse model for neurotrophic keratopathy by approaching the trigeminal nerve from the lateral fornix. Surgical axotomy of the ciliary nerve of the trigeminal nerve was performed in adult BALB/c mice at the posterior sclera. Axotomized, contralateral, and sham-treated corneas were excised on post-operative days 1, 3, 5, 7 and 14 and immunofluorescence histochemistry was performed with anti-β-tubulin antibody to evaluate corneal nerve density. Blink reflex was evaluated using a nylon thread. The survival rate was 100% with minimal bleeding during axotomy and a surgical time of 8±0.5 minutes. The blink reflex was diminished at day 1 after axotomy, but remained intact in the contralateral eyes in all mice. The central and peripheral subbasal nerves were not detectable in the axotomized cornea at day 1 (p<0.001), compared to normal eyes (101.3±14.8 and 69.7±12.0 mm/mm^2^ centrally and peripherally). Interestingly, the subbasal nerve density in the contralateral non-surgical eyes also decreased significantly to 62.4±2.8 mm/mm^2^ in the center from day 1 (p<0.001), but did not change in the periphery (77.3±11.7 mm/mm^2^, P = 0.819). Our novel trigeminal axotomy mouse model is highly effective, less invasive, rapid, and has a high survival rate, demonstrating immediate loss of subbasal nerves in axotomized eyes and decreased subbasal nerves in contralateral eyes after unilateral axotomy. This model will allow investigating the effects of corneal nerve damage and serves as a new model for neurotrophic keratopathy.

## Introduction

The cornea is the most densely innervated tissue in the whole body, [1] supplied by the terminal branches of the ophthalmic division (V1) of the trigeminal nerve as ciliary nerves. Nerve bundles enter the peripheral cornea in a radial pattern, and give off branches that penetrate the Bowman's layer throughout the central and peripheral cornea. These branches then divide and run between the Bowman's layer and the basal epithelium, forming the sub-basal nerve plexus that supplies the overlying corneal epithelium. An intact innervation is necessary for the maintenance of corneal structure and function [2], [3].

Corneal nerves play an important role in regulating corneal sensation, epithelial integrity, proliferation, and wound healing [4], [5]. In addition, more recently, the interaction between corneal nerves and immune cells, [6] and their role in apoptosis [7] and stem cells homeostasis [8] have been reported in humans and animal models, although the exact mechanisms remain unknown. Various neuropeptides and neurotrophins found in the cornea, including substance P (SP), [4] vasoactive intestinal peptide (VIP), [9], [10] calcitonin gene-related peptide (CGRP), [4] α-melanocyte stimulating hormone (α-MSH), [11] nerve growth factor (NGF), [12] brain-derived neurotrophic factor (BDNF) [13] and glial cell-derived neurotrophic factor (GDNF), [13], [14] have anti-apoptotic and anti-inflammatory effects, and promote epithelial wound healing. VIP has been shown to suppress inflammation and to promote the survival of endothelial cells in stored human donor corneoscleral graft. [9], [10] Further,α-MSH regulates the production of pro-inflammatory cytokines and reduces allograft rejection [11].

Corneal nerves may be damaged due to many ocular and systemic pathological conditions, such as acute ocular infection, [6], [15], [16] herpetic eye disease, [17], [18] dry eye syndrome, [19], [20], [21] surgery, [22], [23], [24] diabetes, [25], [26] stroke, [27], [28] and intracranial lesion [29] involving the trigeminal nerve, leading to partial or complete neurotrophic keratopathy (NTK). Subsequent persistent epithelial defects and thinning of the stroma may result in progressive corneal melting and ultimately perforation, potentially leading to permanent vision loss or blindness .[30], [31], [32] Recently, our group has demonstrated that patients with unilateral herpes simplex keratitis (HSK) [18] and herpes zoster ophthalmicus (HZO) [33] showed not only to diminishment of corneal nerves in the affected eye, but also to decreased corneal nerves in the contralateral unaffected eyes. To investigate the mechanisms and timepoint of nerve decrease in contralateral unaffected eye induced by unilateral peripheral nerve damage, reliable animal models are required. Moreover, the implications of contralateral nerve changes on the corneal health remain unknown.

Several animal models of corneal denervation have previously been reported in monkeys, rabbits, rats and mice [3], [7], [34], [35], [36], [37], [38]. These models of NTK have utilized application of hot metal probes, chemical agents or electrolysis through the roof of the mouth or brain skull, with and without the use of stereotactic instruments. However, in these previous NTK models, a success rate of only around 58–90% have been reported, with animals surviving only for 3–6 days, precluding long term follow up. Moreover, while the stereotactic electrolysis approaches from the brain skull or ventral area improved the accuracy of the procedure and the survival rate around 70%, [7], [38] it is extremely invasive, causes neurologic complications and requires specific and expensive setups for stereotactic surgery. Thus, a less-invasive, simple and reproducible peripheral approach is required. To date, a retro-orbital and most direct approach for trigeminal axotomy. It has been thought to be difficult due to the location of the trigeminal nerve posterior to the globe. Herein, we present a novel mouse model of NTK and long-term results of bilateral nerve alterations after unilateral axotomy, using a simple and quick lateral conjunctival approach for trigeminal axotomy. This approach is easy, fast, highly efficient, and has a very low short- and long-term mortality rate, requiring the use of commonly used surgical instruments, accelerating studies on corneal nerve function.

## Methods

### Animals

Six- to 8-week-old male BALB/c mice (Charles River, Wilmington, MA) were used in these experiments. The protocol was approved by the Harvard Medical School Animal Care and Use Committee, and all animals were treated according to the ARVO Statement for the Use of Animals in Ophthalmic and Vision Research.

### Surgical Procedure

**Figure 1:**
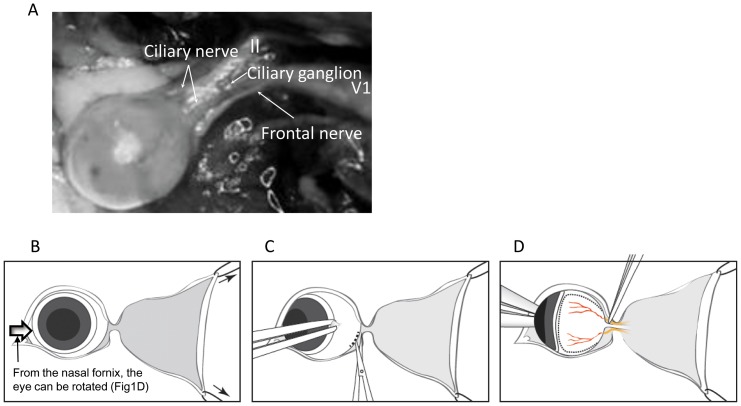
Anatomy of the nerve of the posterior orbit and surgical procedures. A. Anatomy of the branches of the trigeminal nerve (yellow arrows). Two fine ciliary nerve from the ciliary ganglion insert into the posterior sclera aside the optic nerve. B. After lateral canthotomy, two tractional sutures were placed to obtain clear surgical field and allow the eye globe to be dislocated easily. C. The lateral fornix and soft tissue preparation were incised, rotating the eye nasally gently by holding the limbal conjunctiva. D. By gently pushing the nasal fornix using blunt curved forceps, eye globe were rotated to control the eye position, elevate the intraorbital pressure to prevent hemorrhage and enable to see the optic nerve and the branches of the trigeminal nerve. The retinal vessel helped to find the optic nerve and, then the branches of the trigeminal nerve.

Animals were anesthetized with a ketamine (112.5 mg/kg) / xylazine (22.5 mg /kg) / acepromazine (3 mg/kg) mixture. In this surgical procedure, the ciliary nerves of the trigeminal nerve entering the sclera at the posterior globe are axotomized between the sclera and ciliary ganglion. Following periorbital shaving of the fur and disinfection with Povidone Iodine around the lateral skin incision, a 1.5 mm small incision lateral canthotomy was performed. Two tractional 5–0 nylon sutures were placed in the temporally at 45 and 135 degrees in the skin, using straight 1.5” Johns Hopkins clamps (Fig 1B), to allow access to the lateral conjunctiva. The lateral conjunctival fornix was incised circumferentially for 90 degrees with Vannas scissors and conjunctival forceps, taking care not to damage the retro-orbital venous plexus (Fig 1C). Any blood was absorbed using sterile surgical absorber (Sugi, Kettenbach and GmbH & Co. KG, Germany) and the difference of its weight before and after axotomy procedure was measured using a digital balance. After careful preparation of the soft tissues, the lateral rectus muscle and connective tissues with conjunctival forceps, the eye was subsequently rotated nasally about 120 degrees by gently pushing the nasal conjunctival fornix with the blunt tip of eye medium-curved forceps, careful attention not to damage the cornea, exposing the trigeminal nerve. The ciliary nerves of the trigeminal nerve can be identified about 0.3–0.4 mm from the optic nerve insertion. After rotation of the globe, the ciliary nerves of the trigeminal nerve were transected at the posterior sclera close to the optic nerve with sharp forceps under the direct observation (Fig 1D). After cutting the branches of the trigeminal nerve, the tractional sutures were removed and the skin was closed using 8–0 nylon sutures. Antibiotic ointment (AK spore ophthalmic ointment, Akorn Inc., IL, USA) was applied to the sutured area and the treated eye. Two 8–0 nylon sutures for tarsorrhaphy were placed then to reduce the risk of infection and exposure keratopathy. Finally, Fluxinin (0.25 mg/kg body weight for 24 hours) was injected subcutaneously and animals were placed on a heating pad post-operatively. Sham surgery was performed by conducted all steps of the surgical procedure, except the trigeminal axotomy step.

### Corneal Sensation

Corneal sensation of the axotomized and its contralateral unaffected eyes were measured in the central cornea of unanesthesized mice under a dissecting microscope (magnification, ×10) to avoid contact to eyelashes and whiskers, using a 8–0 nylon thread on post-operative days 1, 3, 5, 7 and 14. Results were compared to mice from the sham surgery group.

### Digital Pictures

Digital pictures of the cornea were taken using a microscope at 3, and 7 days after the procedures using the digital camera (Mega Pixels, 9.6 HD, Sharp, Osaka, Japan) to demonstrate changes in transparency, corneal epithelium, corneal edema and neovascularization.

### Immunofluorescent Staining

Corneas from normal eyes, as well as after trigeminal axotomy and sham-surgery were harvested on post-operative days 1, 3, 5, 7, and 14 (at least n = 3/group). Contralateral corneas were excised for both surgical and sham groups. Freshly excised corneas were washed in phosphate-buffered saline (PBS) and fixated in acetone for 15 minutes at room temperature. To block non-specific staining, corneas were incubated in 3% bovine serum albumin (BSA) diluted in PBS for 90 minutes. Corneas were then stained with monoclonal NL637-conjugated anti-β-III tubulin antibody (anti-Neuron-specific β-III Tubulin-NL637, R&D systems Inc. Minneapolis, MN; dilution of 1∶100) at 4C degree overnight. Each step was followed by three thorough washings in PBS for 5 minutes each. Corneal whole mounts were prepared using DAPI mounting medium (Vectashield mounting medium with DAPI; Vector Laboratories, Burlingame, CA). The central and peripheral nerves of the whole thickness corneas were imaged at the z-axis steps of 2 μm using a FV10-ASW confocal microscope (Olympus, Tokyo, Japan).

### Data Analysis and Statistics

**Figure 2:**
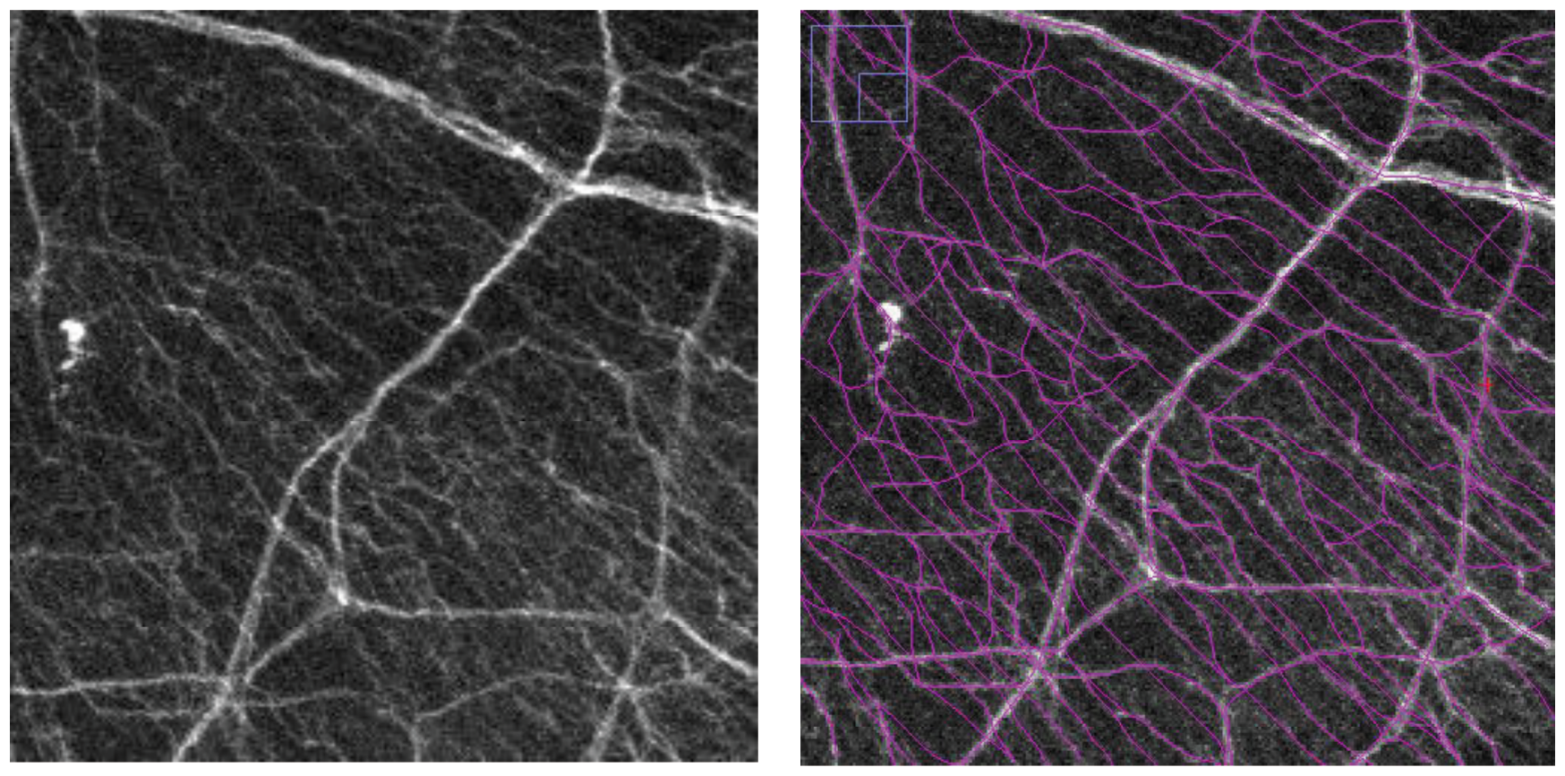
Methods of nerve density calculation using Neuron J. A. Representative stacked images of immunofluorescent histology of normal cornea. B. All nerve branches were traced using Neuron J one by one manually.

Image J 1.45 and Neuron J were used to create the stacked images and calculate the nerve density for subbasal nerves. Neuron J is an Image J plugin software to facilitate the tracing and quantification of elongated image structure (http://www.imagescience.org/meijering/software/neuronj/). All nerve branches of stacked corneal images were traced using Neuron J software after converting the images into 8-bit black and white images (Fig 2). The total length of the traced nerves was quantified by Neuron J for all groups and days. Data were analyzed using statistical analysis software (SSRI Co. Ltd., Tokyo, Japan). The analysis of variance (ANOVA) was used to compare the nerve density between normal, post-axotomy, contralateral, and sham-treated corneas. For each test, differences were considered significant at P value of less than 0.05 and represented as mean +/− SD.

## Results

### Surgical Results

The survival rate was 100% (n =  97 mice) without any systemic complications, including neurologic complications like paralysis. The duration of the surgery was 10 minutes or less (mean 8±0.5 minutes for surgical time, not including anesthesia). The intra-operative bleeding was 0.01 mg or less. Scleral perforation and vitreous prolapsed by sharp forceps occurred in one mouse, which was excluded from the data evaluation.

**Figure 3:**
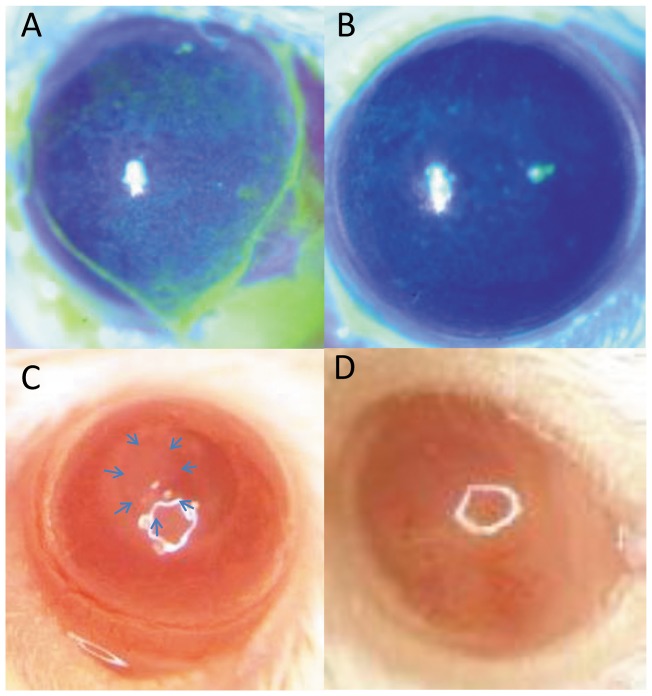
Bilateral superficial epitheliopathy (A–B) and gross pictures of the cornea Day 3 (C) and 7 (D) after trigeminal nerve. Bilateral superficial punctuate keratitis was observed in all cases (A: Axotomized eye, B: its contralateral eye). After trigeminal axotomy, some cases, not all, developed epithelial defect (C), followed by corneal neovascularization (D).

Mild superficial punctuate keratitis (SPK), conjunctival chemosis, and injection in the lateral incision at the fornix were observed in the axotomized eyes from day 1 in all mice (Fig 3A). Conjunctival chemosis and injection resolved at day 5, however SPK continued until day 14. Mild superficial epitheliopathy was observed in its untreated contralateral eyes (Fig 3B). Corneal epithelial defect occurred in around 25% of mice (Fig 3C, blue arrows), which was followed by corneal neovascularization (Fig 3D). Corneal sensation was diminished significantly in all axotomized eyes compared with the control sham group from day 1 after surgery. However, no difference in corneal sensation was observed between the contralateral eyes of axotomized mice as compared to the sham group (Table 1).

**Table 1:** Blink reflex results.

	Day 1	Day 3	Day 5	Day 7	Day14
Axotomized eye	3/3 diminished	3/3 diminished	3/3 diminished	5/5 diminished	5/5 diminished
Contralateral eye	3/3 present	3/3 present	3/3 present	5/5 present	5/5 present

Normal group; 3/3 present, Sham group; 3/3 present.

### Immunofluorescent staining evaluation

**Figure 4:**
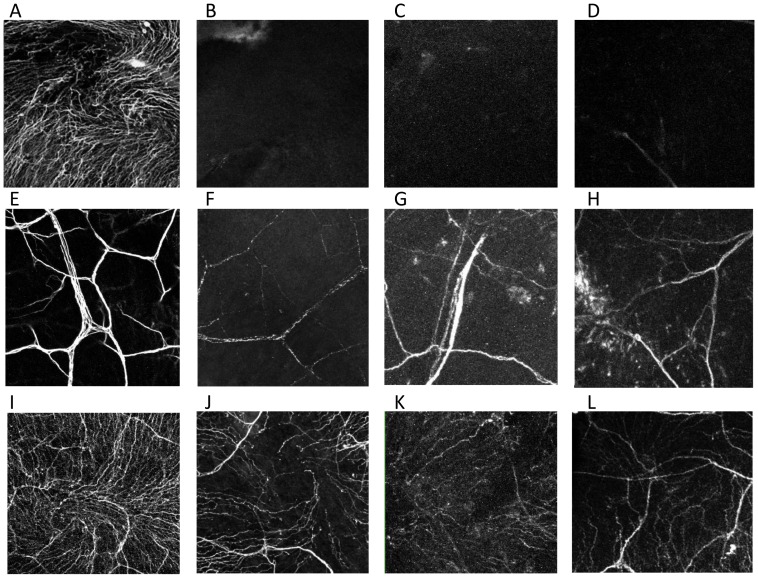
Nerve alteration in the center after axotomy and the contralateral eyes. Representative histological photographs of corneal nerves stained with anti-βIII tubulin FITC-conjugated antibody. Normal subbasal (A) and stromal (E) nerve plexus. Subbasal nerve plexus completely disappeared (B; day 1, C; day 7, D; day 14) and stromal nerve definitely decreased from day1 (F; day 1, G; day 7, H; day 14) and after trigeminal axotomy. The nerve of the contralateral eye decreased from day 1 (J; day 1, K; day 7, L; day 14).

**Figure 5:**
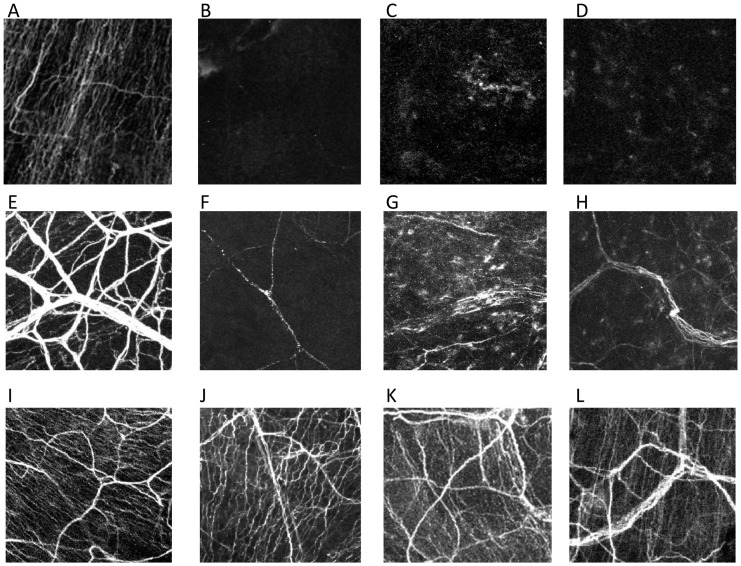
Nerve alteration in the peripheral cornea after axotomy and the contralateral eyes. Representative histological photographs of corneal nerves stained with anti-βIII tubulin FITC-conjugated antibody. Normal subbasal (A) and stromal (E) nerve plexus.Subbasal nerve plexus completely disappeared (B; day 1, C; day 7, D; day 14) (A) and stromal nerve definitely decreased (B) from day 1 (F; day 1, G; day 7, H; day 14) after trigeminal axotomy. The nerve of the contralateral eye did not change (J; day 1, K; day 7, L; day 14).

**Figure 6:**
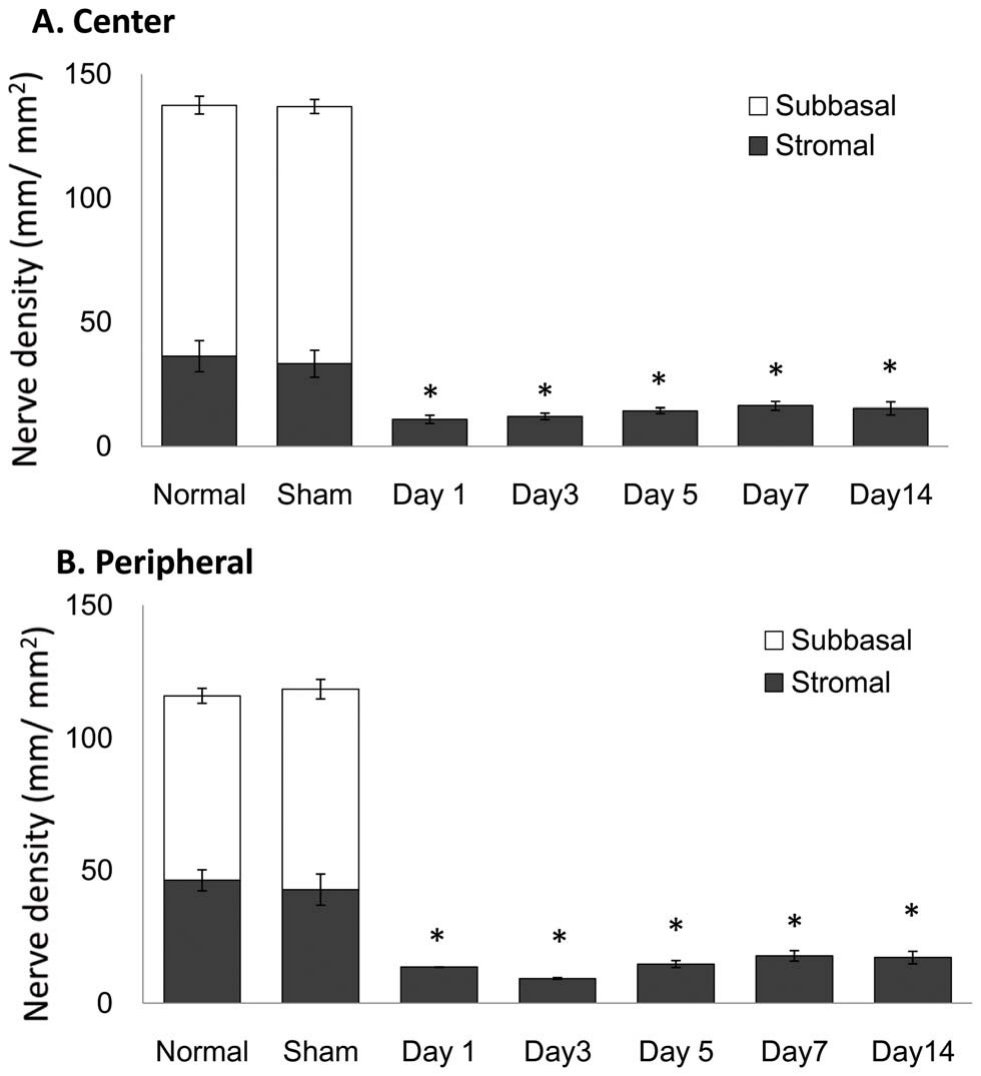
Nerve density alteration after axotomy. The nerve density significantly decreased from day 1 in axotomized eye in the central (A) and peripheral cornea (B) (ANOVA, both P<0.001).

Corneal nerves (Fig 4 and 5) decreased immediately after trigeminal axotomy (Fig 4B–D, F–H and Fig 5B–D, F–H) as compared to normal (Fig 4A, E and Fig 5A, E) and sham-treated eyes (Fig 4I and Fig 5I). The subbasal nerve plexus of the axotomized eyes were completely diminished from post-operative day 1, even in mice without epithelial defects (Fig 4B–D). Central subbasal nerves density (Fig 6A) were reduced from day 1 (Fig 4 B, 0.0±0.0 mm/mm^2^), compared to normal (Fig 4A, 101.3±8.5 mm/mm^2^, P<0.001) and sham-treated eyes (Fig 4I, 103.8±5.5 mm/mm^2^, P<0.001), and remained completely absent until day 14. Further, there was no significant difference in central subbasal nerve density between normal and sham-treated eyes (P = 0.48). In the periphery (Fig 6B), compared to normal (Fig 5A, 69.7±7.0 mm/mm^2^, P<0.001) and sham-treated eyes (Fig 5I, 75.8±5.9 mm/mm^2^, P<0.001), subbasal nerves were diminished in the axotomized eye from day1 (Fig 5B, 0.0±0.0 mm/mm^2^) and remained completely absent (Fig 5C–D, 0.0±0.0 mm/mm^2^) until day 14. There was no significant difference in peripheral subbasal nerve density between normal and sham-treated eyes (P = 0.52).

Stromal nerves significantly decreased from day 1 post-axotomy, lost their plexiform structure, appeared fragmented, and did not change over the follow-up period after day 1 (Fig. 4F–H and 5F–H). Stromal nerves (Fig 4E–H, 5E–H) were reduced from day 1 (center; 10.9±1.7 mm/mm^2^, peripheral; 13.6±0.1 mm/mm^2^), compared to normal (center; 36.3±6.3 mm/mm^2^, P<0.001, peripheral 46.3±4.0 mm/mm^2^, P<0.001) and sham-treated eyes (center 33.2±5.5 mm/mm^2^, P<0.001, peripheral 42.8.2±5.9 mm/mm^2^, P<0.001). Stromal nerve density remained low and did not change significantly until day 14 (center 15.3±2.6 mm/mm^2^, peripheral 17.3±2.3 mm/mm^2^).

**Figure 7:**
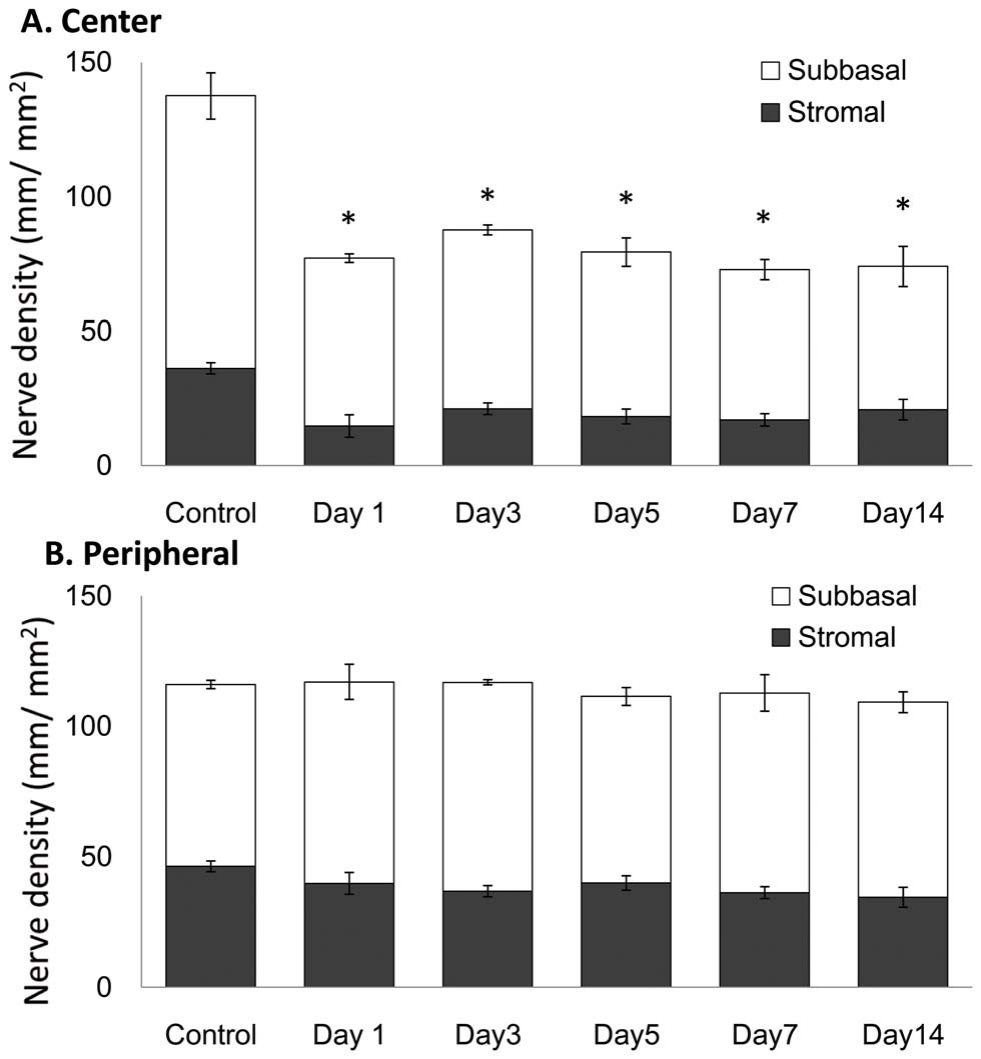
Nerve density alteration after axotomy in its contralateral eye. A. The nerve density in the contralateral eye significantly decreased from day 1 (P<0.001). B. The nerve density in the peripheral cornea did not significantly change in its contralateral eye (P = 0.82).

Surprisingly, subbasal corneal nerves in the corneal center of the contralateral eye (Fig 4 J–L and Fig 7A) were reduced from day 1 (77.3±6.9 mm/mm^2^), compared to normal eyes (101.3±8.5 mm/mm^2^, P<0.001) and sham-treated eyes (103.8±5.5 mm/mm^2^, P<0.001), and slowly decreased until day 14 (Fig 4L, 53.2±7.4 mm/mm^2^, P<0.001). However, contrary to the central cornea, no alterations could be observed in the subbasal nerves of the contralateral peripheral cornea (Fig 5J–L and Fig 7B, P = 0.82). Stromal nerves in the corneal center of the contralateral eyes were reduced from day 1 (10.9±1.7 mm/mm^2^) compared to normal eyes (36.2±6.2 mm/mm^2^, P = 0.005) and sham-treated eyes (33.2±5.5 mm/mm^2^, P = 0.008) and remained low until day 14. Stromal nerve in the peripheral cornea of the contralateral eyes did not change.

## Discussion

The cornea as the most densely innervated tissue of the body, is supplied by the terminal branches of the ophthalmic division of the trigeminal nerve as ciliary nerves [1], [2]. Sensory innervation of the cornea from the trigeminal nerve is important for perceiving corneal sensation, as well as for epithelial integrity, epithelial proliferation, wound healing, and avoiding injury [39], [40]. Corneal nerve dysfunction forms the pathophysiologic basis of ocular disease, causing considerable morbidity such as NTK [41]. Many corneal and neurological diseases may result in NTK, albeit with various degrees of severity. These include, but are not limited to, ocular infections, [6], [15], [16] herpetic eye disease, [17], [18] dry eye syndrome, [19], [20], [21] surgery, [22], [23], [24] diabetes, [25], [26] stroke, [27], [28] and intracranial lesion [29]. NTK as a result of these diseases can often result in persistent epithelial defects, stromal thinning, progressive corneal melting, and ultimately perforation and permanent vision loss or blindness. [30], [31], [32] Further, while corneal perforation or corneal scaring as a result of NTK may requrie corneal transplantation, these transplants often have difficulty with epithelial wound healing and have a very high rate of graft rejection. Thus, it is necessary to investigate the effect of corneal nerve damage on the corneal pathophysiology. Our current animal model presented herein, is an acute nerve injury mode, resulting in neurotrophic keratopathy, and may allow studying corneal nerve function and the consequences of lack thereof in more depth.

Several previous animal models for NTK have previously been described and summarized in Table 2. The animal model for NTK is essential to investigate the pathophysiology of NTK and nerve change in the contralateral eye and to develop new treatment of NTK. Singelman and Friedenwald reported a new approach to denervate the trigeminal ganglion using diathermy electrode, resulting in neuro-paralytic keratitis of the rat [34]. Further, Schimmelpfennig and Beuerman controlled sensory denervation of the rabbit cornea by performing radiofrequency thermocoagulation through the soft palate in rabbits by entering the open mouth with hypodermic needle [36]. Nagano et al were the first to report the stereotactic approach from the brain to produce NTK in rat, [37] followed by the reports by Wong et al. that used the ventral approach [38] and Ferrari et al. from the brain skull [7]. However, the ventral approach had a mortality rate of 7% (4/56) and in the recent report by Ferrari et al. the mortality was not stated. The common target of all of these reports was the trunk or ganglion of the trigeminal nerve in the brain, which caused the neurologic complications such as paralysis, varying the incidence rate from 7% to 40%. A previous intraorbital approach has been described by Gallar et al. [3] using injection of a mixture of ethanol and capsaicin into the retro-orbital space. This pharmacologic approach, while novel and original, is highly inflammatory and damages other soft tissues and optic nerve. In contrast to the previous models of NTK, our lateral conjunctival approach to trigeminal axotomy has several major advantages, including a high survival rate of animals (100%) allowing long-term follow-up, lack of interference with central nervous system, no surgical effects on the contralateral eye, minimal invasiveness to animals and a short surgical time, no need for specialized and expensive stereotactic equipment, high reproducibility, and a high success rate due to the ability to perform axotomy under direct visualization.

**Table 2:** Literature review of NTK animal models.

Author (year)	Animal	Approach	Way of damage	Merit	Problem
Ferrari (2011) [7]	Mouse	Brain skull stereotactic	Electrolysis	70% success 90% survival rate without neurologic complications	Blind procedure Need specific expensive instrument
Wong (2004) [38]	Rat	Ventral stereotactic	Electrolysis	100% success 93% survival	Blind procedure V2 damage
Nagano (2003) [37]	Rat	Brain skull stereotactic	Coagulation	80% success	Brain damage
Gallar (1995) [3]	Rabbit	Retrobulbar injection	99% Ethanol 1% Capsaicin	High success	Severely damage retro-bulbar tissues
Schimmelpfennig (1982) [36]	Rabbit	Mouth	Needle	Mortality rate <5%	Blind procedure
Keen (1982) [35]	Mouse	Subcateneous injection in neonate	Capsaicin Ethanol	65% success	Possible damage retro-bulbar tissue
Sigelman (1954) [34]	Rat	Mouth	Coagulation	60% success	High fatality
Current method	Mouse	Lateral conjunctival fornix	Mechanical	95.8% success* 100% survival rate	

* Complete subbasal nerve loss was observed in 23 corneas out of 24 (95.8%) In one cornea (4.2%), there was partial residual subbasal nerve observed at Day 7.

NTK: neurotrophic keratopathy.

Although the corneal innervation is believed to be mediated along a unilateral ophthalmic nerve pathway, [42] https://bay166.mail.live.com/mail/ – bilateral nerve alterations after unilateral nerve damage have recently been reported by our group in patients with herpes simplex keratitis (HSK) [18] and more recently in herpes zoster ophthalmicus (HZO). [33] In both patients with HSK and HZO, the loss of corneal sensation was reported to be correlated with profound diminishment of the subbasal nerve plexus. Although HSK has been reported to be bilateral in 10–20% of cases, HZO is clearly a unilateral disease and our findings on bilateral nerve changes had been surprising.

Contralateral effects after unilateral diseases or experimental models have previously been reported in the literature [43]–[48]. Keijer et al. and Simard-Lebrum et al. compared bilateral tear production in patients with unilateral HSK and found no difference between the basal tear secretion of affected and unaffected eyes in these patients, although both eyes demonstrated significantly lower rates than normal subjects [43], [44]. Further, diurnal fluctuation of intraocular pressure (IOP), is known to be symmetrical in normal eyes and eyes with glaucoma, with a fairly high concordance [45], [46]. In addition, in a capsaicin-induced neurogenic inflammation model, Gonzales et al. demonstrated that while aqueous humor protein level were different at 30 minutes after treatment, both treated and contralateral eyes showed similar levels 5 hours after treatment [48]. We would like to note that none of the previous experimental animal studies evaluated nerve alterations in the contralateral eyes after unilateral procedures of trigeminal axotomy [2]–[5], [7], [8], [34]–[39].

The bilateral effects after unilateral lesions have been observed in numerous experimental and clinical paradigms outside the eye [49]. The effects range from pain development to inflammation, from nerve changes to muscle fibrosis, and from altered gene expression to tissue remodeling [49]–[54]. The most illustrating examples of “mirror” effects are strictly symmetrical inflammatory changes in rheumatologic diseases [55] and pain development in symmetric skin areas [56]. The exact mechanisms behind the contralateral changes are unclear, even though the presence of bilateral effects after nerve lesions is well-known for more than 45 years in non-ocular studies [57].

There is broad agreement that central nervous system pathways are responsible for these midline-crossing effects, documented also by affection of contralateral undamaged neurons after peripheral nerve lesions and the neurogenic topographical precision [49], [55], [56], [58], [59]. Nerve degeneration in the contralateral side of the body (similar to our findings in the cornea) was very recently demonstrated in a model of unilateral muscle overuse with profound axonal degeneration bilaterally [54]. Also, unilateral shingles has been shown to induce nerve alterations in the unaffected contralateral skin, [60] and unilateral surgical denervation resulted in reduced innervation and sensory function in the contralateral hind paws of animals [53]. Several established anatomical pathways may account for the neuronal transmedian signaling that in most parts of the neuraxis mediates bilaterality via a commissural system [49], [61]. In addition, thalamus and rostral ventral medulla represent anatomical structures involved in bilateral control of descending pathways, [62] and trigeminal (including corneal) fibers indeed innervate bilateral brainstem areas and traverse between trigeminal nuclei of both sides [63], [64].

Several potential biochemical mechanisms have been suggested to mediate the neurogenic bilateral effects, including release of inflammatory mediators from neurons, such as TNF-a, IL-1b, IL-10, MCP-1, neurotensin, nitric oxide, CGRP, or substance P [49], [62], [65], [66]. Other studies demonstrated the functional activity, metabolism and genomic expression in contralateral side in animal models [67], [68], [69]. However, a final proof is lacking in all models so far. Along these lines, our minimally invasive and anatomically precise model of ciliary nerve damage might be a useful experimental paradigm to follow these rather universal mechanistic questions in more detail.

In the current study, the nerve density of contralateral eye was consistently around 60 mm/mm^2^ (40% decrease from normal level) through the follow up periods. We were not able to observe epithelial defect, neovascularization, nor the reduced corneal sensation in any of these contralateral eyes, all of which developed in axotomized eyes. These findings are in accordance with our previous clinical studies in HSK and HZO [18], [33], which demonstrated that abnormal sensation (and potentially other functions) is only noted, only after significant diminishment in corneal nerves of greater than 50%. Moreover Gallar et al. recently showed that heat sensation was decreased in the contralateral eyes of HSK patients [70]. Finally, they did observe a slight decrease in cornealsensitivity in contralateral eyes of patients with HSK, although the results were not statistically significant. It is a general phenomenon that the contralateral effects are less pronounced than ipsilateral ones, [49] such as reduced pain areas in hyperalgesia models [52] or diminished inflammation [71]. Correspondingly, contralateral nerve degeneration in the cornea in our study was less prominent and restricted to the more distal parts of the nerves (central cornea). The difference of nerve fiber structure between the center and the periphery could also explain why the nerves in contralateral eyes are affected centrally. While some of the central nerves include unmyelinated nerver fibers (C-type), peripheral nerves are myelinated (A-delta type) [1]. Fine nerve fibers without myelin sheath in the axon have been shown to degenerate faster than the nerve with myelin sheath from the distal area [72]. Further, Keijser et al. postulated that some sensory nerve endings of the contralateral cornea may become damaged due to bilateral alteration of tear composition and volume they found in unilateral HSV patients [43]. While we did observe mild contralateral epitheliopathy in our experiments, further studies may determine the detailed relation between the location and potential causal relationships between epitheliopathy and subbasal nerve loss.

The current surgical procedure successfully resulted in the diminishment and complete loss of subbasal nerve plexus and corneal sensation as early as at day 1 after axotomy. However, despite significant reduction of stromal nerves, stromal nerve fragments were observed by immunohistochemistry. Similar findings can be observed in the study by Ueno et al. after stereotactic ablation of the trigeminal nerve, where apparent stromal nerves could be observed 7 days after surgery [8]. Although most of the previous studies in animal models of neurotrophic keratopathy did not use immunohistochemistry, histochemical studies in some reports [8], [36] showed degenerative alterations, such as fragmented nerves after axotomy. The mammalian cornea has previously been shown to be innervated by modest sympathetic nerves. [73] Further, Marfurt et al [74] have demonstrated that rat cornea receive modest parasympathetic nerves, which remained after both sensory and sympathetic denervation. Interestingly, fewer than one-third of all ciliary ganglion neurons are present in the main ciliary ganglion, with most neurons being located in accessory ciliary ganglia within the optic nerve sheath plexus [75]. Thus, our trigeminal axotomy model, while achieving sensory denervation, does likely not lead to complete denervation of parasympathetic and potentially sympathetic nerve fibers. Moreover, previous studies demonstrated βIII-tubulin expression in proliferating Schwann cells after sciatic nerves transection or damages in rat models [76], [77]. Thus, it is conceivable that Schwann cells in the cornea may similarly upregulate βIII-tubulin. Finally, although less likely, potential collateral innervation into the cornea may exist from facial nerves [Dr. Carlos Belmonte, personal communication].

In conclusion, compared to previously reported models, our trigeminal axotomy mouse model is highly effective, easy, fast, less invasive, and has a superior survival rate. This model will enable us to investigate the effects of corneal nerve damage not only short-term, but also long-term and serves as a unique model for NTK. Using this less-invasive model, we also demonstrated the immediate decrease in subbasal nerve density in the center of contralateral corneas.
